# Adsorption of Quercetin on Brown Rice and Almond Protein Matrices: Effect of Quercetin Concentration

**DOI:** 10.3390/foods11060793

**Published:** 2022-03-09

**Authors:** Mirela Kopjar, Ivana Buljeta, Ina Ćorković, Anita Pichler, Josip Šimunović

**Affiliations:** 1 Faculty of Food Technology, Josip Juraj Strossmayer University, F. Kuhača 18, 31000 Osijek, Croatia; ivana.buljeta@ptfos.hr (I.B.); ina.corkovic@ptfos.hr (I.Ć.); anita.pichler@ptfos.hr (A.P.); 2 Department of Food, Bioprocessing and Nutrition Sciences, North Carolina State University, Raleigh, NC 27695-7624, USA; simun@ncsu.edu

**Keywords:** quercetin, almond protein matrix, brown rice protein matrix, HPLC, antioxidant activity, DSC, FTIR-ATR

## Abstract

Plant-based proteins are very often used as carriers of different phenolic compounds. For that purpose, complexation of quercetin with almond and brown rice protein matrices was investigated. The amount of protein matrices was constant, while the concentration of quercetin varied (1 mM, 2 mM or 5 mM) during complexation. Dried complexes were investigated for quercetin amount (HPLC analysis) and antioxidant activity (DPPH, FRAP and CUPRAC methods). Additionally, complexation was proven by DSC and FTIR-ATR screening. An increase in the concentration of quercetin in the initial complexation mixture resulted in the increase in the adsorption of quercetin onto protein matrices. For the brown rice protein matrices, this increase was proportional to the initial quercetin concentration. Adsorption of quercetin caused the change in thermal stability of microparticles in comparison to corresponding protein matrices that have been proven by DSC. FTIR-ATR analysis revealed structural changes on microparticles upon adsorption of quercetin.

## 1. Introduction

A growing area of interest in the field of polyphenols is their interactions with other components present in the food matrix, including proteins, carbohydrates and lipids [1]. Proteins as building blocks have both nutritional and functional properties and are an important dietary source of amino acids [2]. As an outcome of interactions between polyphenols and proteins, various complexes can be formed, which consequently cause changes in antioxidant properties of polyphenols and affect the functional, structural and nutritional properties of proteins [3]. Flavonoids are an important subgroup of polyphenols and quercetin, as a member of the flavonol subclass of flavonoids is found in various fruits, vegetables and tea [4]. The intake of quercetin is related to the assembly of beneficial health properties such as antioxidant, anti-inflammatory and anti-viral properties, improvement in cardiovascular health [5] and reducing the intensity of the symptoms and negative predictors of COVID-19 [6], which is a hot topic nowadays. Its anticancer effect was also established in numerous studies [7,8]. For the aforementioned reasons, the involvement of quercetin in the human diet is strongly recommended and it is proposed as an excellent ingredient for functional foods [9]. On the other hand, its broader application is limited because of insufficient water solubility and chemical instability. The application of an adequate delivery system is one way to achieve stabilization and improvement of the health benefits of quercetin. The “Delivery by design” approach leads to the expansion of the search for effective delivery systems of bioactives in order to encapsulate them; on the one hand for their protection from environmental factors and on the other hand to control their release under defined conditions. Possibilities for the application of delivery systems can be various from food, agrochemical, pharmaceutical, cosmetic to personal care industries [10]. There are various types of delivery systems, and each one has specific advantages and disadvantages; thus, the selection should be based on the field of utilization [11]. Both animal-based and plant-based protein matrices were used for complexation with polyphenols. The most used animal-based protein matrices were whey, gelatin, milk proteins, bovine serum albumin [12,13,14,15,16]. Applications of plant-based protein matrices are gaining higher attention and proteins from different sources such as sunflower, legume seed, corn kernels, quinoa, wheat, rice, peas, hemp, almond and pumpkin [17,18,19,20,21,22,23,24,25,26,27,28] have been used for the preparation of complexes which can be further used as functional food additives. As a result of consumers’ awareness of the importance of a healthy and balanced diet, functional foods are becoming more popular. In addition, convenience is becoming a very important element in the selection of foods. A very busy and mobile lifestyle, the search for simple meal preparation and easier consumption, healthy snacking options in as well as outside of homes are also some of the emerging trends among consumers [3,17,19,29] that need to be addressed by the food industry.

The aim of this research was to prepare microparticles from brown rice or almond protein matrices and quercetin in order to investigate whether different concentrations (1 mM, 2 mM or 5 mM) of quercetin in the initial mixture had an impact on its adsorption on proteins. Determination of quercetin concentration using high-performance liquid chromatography (HPLC) and antioxidant activities of prepared microparticles were performed. Additionally, DSC and FTIR-ATR screening of microparticles were performed. DSC analysis was conducted to evaluate the effect of quercetin interactions with proteins on the thermal stability of protein matrices, while screening of IR spectra was performed as proof that interactions between quercetin and protein matrices occurred.

## 2. Materials and Methods

### 2.1. Materials

Almond protein matrix was purchased from Raab Vitalfood GmbH (Rohrbach, Germany) and brown rice protein matrix was from Kernnel premium (Zagreb, Croatia). Quercetin, trolox and 2,2-diphenyl-1-picrylhydrazyl (DPPH) were obtained from Sigma-Aldrich (St. Louis, MO, USA). Sodium carbonate was obtained from Kemika (Zagreb, Croatia). Neocuproine, copper (II) chloride and 2,4,6-tri(2-pyridyl)-s-triazine (TPTZ) were obtained from Acros Organics (Geel, Belgium). Orthophosphoric acid (HPLC grade > 85%) was obtained from Fisher Scientific (Loughborough, UK), while methanol (HPLC grade) was purchased from J.T. Baker (Deventer, The Netherlands). Iron (III) chloride hexahydrate, ethanol, sodium acetate and ammonium acetate were purchased from Gram-mol (Zagreb, Croatia).

### 2.2. Preparation of Protein/Quercetin Microparticles

The microparticles were formulated by the complexation of protein matrices (constant amounts; 5%) with 20 mL of quercetin ethanol solution (1 mM, 2 mM or 5 mM). Two protein matrices were used (both are usually used as dietary supplements), brown rice with approximately 85% of proteins (7.7% of carbohydrates and 5.1% of lipids), and almond with approximately 50% of proteins (fibers 17%, 9% of carbohydrates and 11% of lipids). The preparation method was adapted from other studies [21,27,28]. The protein matrix was weighed and added to the quercetin solution. In order to prepare protein/quercetin microparticles, obtained mixtures were mixed on a magnetic stirrer for 15 min at room temperature. During that time, part of quercetin was adsorbed onto the protein matrix and the other part remained in the solution. Afterward, well-homogenized mixtures were centrifuged for 15 min at 4000 rpm in order to remove the quercetin that did not adsorb onto the protein matrix and remained in the supernatant. The supernatant was discarded and the wet–solid phase that represented adsorbed quercetin onto protein matrix was collected. After air-drying, protein/quercetin microparticles were obtained in the form of dried powder.

### 2.3. Extraction of Quercetin from Protein/Quercetin Microparticles

Quercetin was extracted from obtained protein/quercetin microparticles. Microparticles were weighted (0.15 g), 10 mL of acidified methanol (methanol:HCl ratio was 99:1) was added and the obtained mixture was well homogenized. Extraction was conducted at room temperature for 24 h. After that time, mixtures were filtered to obtain clear extracts which were immediately utilized for the determination of the amount of quercetin and antioxidant activities.

### 2.4. Reverse-Phase High Performance Liquid Chromatography (RP-HPLC)

A RP-HPLC system (1260 Infinity II; Agilent technology, Santa Clara, CA, USA) was used for the evaluation of the amount of quercetin. The system was equipped with a DAD (diode array) detector, a quaternary pump and a column (poroshell 120 EC-C 18; 4.6 × 100 mm, 2.7 µm). Two mobile phases were used; mobile phase A was orthophosphoric acid (0.1%) and mobile phase B was methanol (100%). The gradient that was applied for separation was described in previous studies [27,28]. The injection volume of the extract was 10 µL, under the flow rate of 1 mL/min at room temperature. A calibration curve for quercetin was constructed in the range from 5 to 150 mg/L. UV/Vis spectra was screened in the range from 190 to 600 nm, and quercetin was determined at 360 nm. Duplicate evaluations were conducted.

### 2.5. Antioxidant Activity

DPPH, CUPRAC and FRAP methods were utilized for the evaluation of antioxidant activities of microparticles extracts. Details for these methods were previously given by Buljeta et al. [30]. Assays were performed in triplicate and results were presented as micromoles of Trolox equivalent per 100 g of sample (µmol TE/100 g).

### 2.6. Differential Scanning Calorimetry (DSC)

For the DSC scanning of microparticles, a differential scanning calorimeter (Mettler Toledo 822, Mettler Toledo, Greifensee, Switzerland) was applied. In a 40 µL aluminum pan, 7 ± 0.2 mg of microparticles was weighed. The aluminum pan was covered and then inserted into the oven of the DSC. Screening of microparticles was performed from 25 °C to 140 °C. Firstly, samples were left for 4 min at 25 °C. Afterwards, the temperature was increased at a rate of 5 °C/min up to 140 °C, where samples were also left for 4 min. Duplicate screenings were conducted.

### 2.7. Fourier-Transform Infrared Spectroscopy-Attenuated Total Reflectance (FTIR-ATR)

The IR spectra of protein matrices and protein matrices loaded with quercetin were recorded using FTIR-ATR (Cary 630 FTIR spectrometer, Agilent Technology, Santa Clara, CA, USA), equipped with software MicroLab Expert. Samples were screened in the interval from 4000 cm^−1^ to 600 cm^−1^.

### 2.8. Statistical Analysis

STATISTICA 13.1 (StatSoft Inc., Tulsa, OK, USA), the software program, was utilized for analyzing the obtained results. Variance analysis (ANOVA) and Fisher’s least significant difference (LSD) with significance defined at *p* < 0.05 were selected for statistical evaluation of the results, which were presented as mean value ± standard deviation.

## 3. Results

### 3.1. Quercetin Amount and Antioxidant Activity of Protein/Quercetin Microparticles

The amounts of quercetin and antioxidant activities of protein/quercetin microparticles are given in Table 1. Comparison of almond protein/quercetin (AP/Q) microparticles and brown rice/quercetin (RP/Q) microparticles showed that RP/Q microparticles had a higher amount of quercetin than AP/Q microparticles prepared with the same initial concentration of quercetin, indicating that the brown rice protein matrix had a higher affinity towards quercetin. Additionally, it can be observed that an increase in concentration of quercetin in the initial solution for complexation, resulted in an increase in the amount of quercetin. Results were compared in order to investigate whether this increase in quercetin amount was proportional to the initial concentration of quercetin. Amounts of quercetin on AP/Q microparticles were 60.18, 98.38 and 196.34 mg/100 g for AP/Q_1, AP/Q_2 and AP/Q_5 (i.e., 1 mM, 2 mM and 5 mM of quercetin in initial solution), respectively. Results indicated that with the double increase in the initial quercetin, the resulting concentration increase was 1.6 times, and for the five times increase in the initial quercetin concentration, the resulting increase was 3.7 times higher. For RP/Q microparticles, a different trend was observed. Amounts of quercetin on microparticles were 108.24, 226.50 and 506.98 mg/100 g for RP/Q_1, RP/Q_2 and RP/Q_5 (i.e., 1 mM, 2 mM and 5 mM of quercetin in initial solution), respectively. Results indicated that with a double increase in initial quercetin concentration, the increase was 2.1 times and with a five times increase in initial quercetin concentration, the resulting increase was 4.7 times higher. Interesting data were obtained by comparing the amount of the quercetin on microparticles to the initial amount of quercetin, i.e., calculating the adsorption efficiency of protein matrices towards the quercetin. For the almond protein matrix, as was expected, a lower efficiency was determined than for the brown rice protein matrix. Even though the quercetin amount increased on A/Q microparticles with the initial quercetin amount, a decrease in adsorption efficiencies were observed; 20%, 16.3% and 13% for AP/Q_1, AP/Q_2 and AP/Q_5, respectively. For RP/Q microparticles, a slightly different trend was obtained, i.e., for RP/Q_1 and RP/Q_2, adsorption efficiencies were 36.8% and 37.5%, and for RP/Q_5, 33.6%.

For the evaluation of antioxidant activities of microparticles, three methods were selected: DPPH, FRAP and CUPRAC methods. Values for antioxidant activities obtained with all methods followed the amount of quercetin, i.e., an increase in quercetin amount caused an increase in antioxidant activity. However, a trend of the proportional increase in antioxidant activity with the quercetin amount was not observed for all methods. DPPH antioxidant activities for AP/Q microparticles were from 29.38 to 32.18 µmol TE/100 g, while for RP/Q, microparticles values were slightly higher, from 31.12 to 40.40 µmol TE/100 g. Values of antioxidant activities were much higher with the CUPRAC method and they ranged from 106.39 to 192.75 µmol TE/100 g for AP/Q microparticles and from 84.30 to 414.52 µmol TE/100 g for RP/Q microparticles. With the FRAP method, the lowest values of antioxidant activities were obtained. For AP/Q microparticles, they ranged from 0.66 to 2.34 µmol TE/100 g, and for RP/Q microparticles, from 1.20 to 8.35 µmol TE/100 g.

### 3.2. Temperature of Denaturation of Protein Matrices and Protein/Quercetin Microparticles

The results of the DSC determination of denaturation temperatures of protein matrices and protein/quercetin microparticles are presented in Table 2. Both protein matrices had similar values of denaturation temperature at 85.25 °C. When comparing the obtained microparticles, AP/Q microparticles had lower denaturation temperatures (around 83.5 °C) than the corresponding protein matrix, while RP/Q microparticles had it higher (from 85.78 to 86.72 °C). Additionally, a difference in the enthalpy of denaturation was observed. For RP/Q complexes the enthalpy of denaturation increased for 3 J/g, while for AP/Q complexes, the increase was for 1.5 J/g.

### 3.3. FTIR-ATR Spectra of Protein Matrices and Protein/Quercetin Microparticles

**Figure 1:**
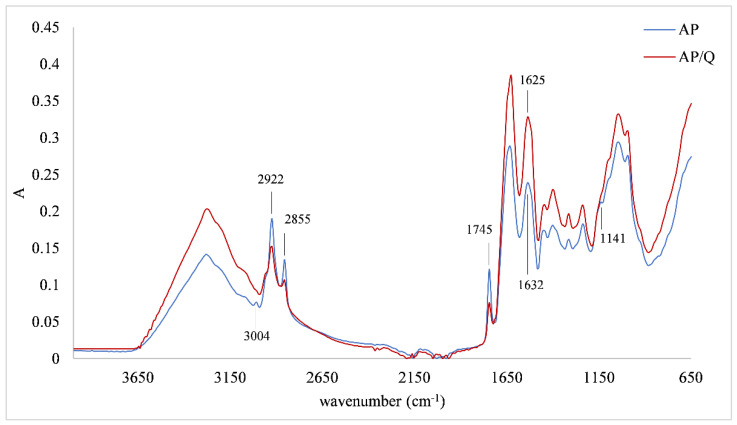
IR spectra of almond protein matrix (AP) and almond protein/quercetin microparticles (AP/Q).

Changes in IR spectra that were obtained by FTIR-ATR screening of protein/quercetin microparticles prepared with different initial concentrations of quercetin were the same, so only one IR spectra of microparticles was presented (the one obtained with 5 mM of quercetin). Figure 1 represents the IR spectra of the almond protein matrix and almond protein/quercetin microparticle. Comparing those two spectra, changes in protein structure after the adsorption of quercetin were established. In two regions, one from 3500 cm^−1^ to 3000 cm^−1^ and another one from 1650 cm^−1^ to 600 cm^−1^, the intensity of the protein spectra was lower than for the microparticle. However, in the region from 3000 cm^−1^ to 2800 cm^−1^ and for the band at 1745 cm^−1^, a reverse tendency was observed. The region from 3500 cm^−1^ to 3000 cm^−1^ can be assigned to amide A, N-H stretching coupled with hydrogen bonding. Additionally, in this region, the band at 3004 cm^−1^ assigned to the C-H bond disappeared after quercetin adsorption. In the region from 3000 cm^−1^ to 2800 cm^−1^, two bands were detected, one at 2922 cm^−1^ and another one at 2855 cm^−1^ both assigned to CH_2_ stretching. A band at 1745 cm^−1^ can be assigned to the C=O band of polysaccharides. Additionally, a change in the Amid I structure of protein occurred. A band at 1632 cm^−1^ shifted to 1625 cm^−1^ after the adsorption of quercetin. One additional change caused by quercetin adsorption was a loss of shoulder at 1141 cm^−1^ (assigned to C-O band of oligosaccharides) on the protein matrix.

**Figure 2:**
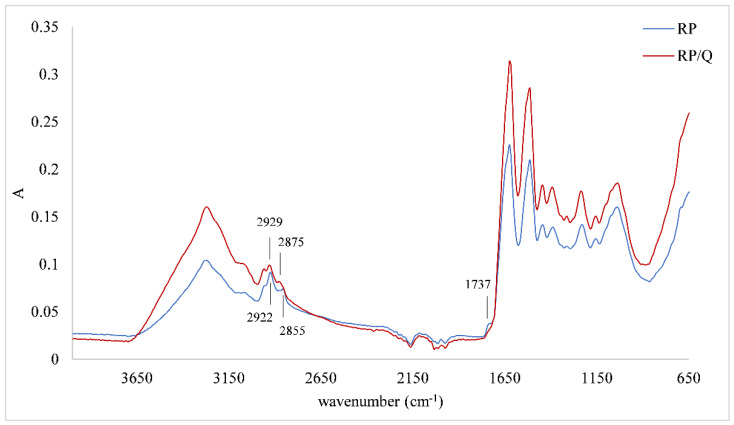
IR spectra of brown rice protein matrix (R) and brown rice protein/quercetin microparticles (RP/Q).

Figure 2 represents IR spectra of other set of samples, i.e., the brown rice protein matrix and brown rice protein/quercetin microparticle. Even though results of the amount of adsorbed quercetin showed that RP/Q microparticles had a higher amount of this phenolic, structural changes were not so pronounced as for AP/Q microparticles. Two bands that were detected at 2922 cm^−1^ and 2855 cm^−1^, both assigned to CH_2_ stretching, after adsorption of quercetin, shifted to 2929 cm^−1^ and 2875 cm^−1^. Another change was detected at 1737 cm^−1^ (assigned to C=O band of polysaccharides) that disappeared after the adsorption of quercetin.

## 4. Discussion

There have been many different instrumental techniques used for the characterization of protein/phenolics complexes [31]. In this study, we applied HPLC, DSC and FTIR-ATR analyses for evaluation of the adsorption of quercetin on selected protein matrices. Interactions that are created between phenolics and proteins upon their complexation depend on the structure of both compounds, as well as complexation conditions [32,33]. Similar protein matrices for the adsorption of different phenolics were used in other studies. Adsorption of glucosyl-hesperidin on pea, almond, pumpkin and brown rice protein matrices revealed that glucosyl-hesperidin was determined in the highest amount on pea protein microparticle, followed by almond, brown rice and in the lowest amount on pumpkin protein microparticles [27]. For the adsorption of cinnamic acid on pea, almond and pumpkin protein matrices, a different trend was observed; hence, cinnamic acid had the highest affinity for pumpkin and the lowest for almond protein matrices [28]. The investigated protein matrices differ in the protein content; pea and brown rice protein matrices had 85% of protein content, while the almond and pumpkin had 50%. Other organic molecules such as polysaccharides can be incorporated in different types of protein matrices [3,27,28,31,34], consequently having an effect on the adsorption of phenolics onto them. As a result of the encapsulation of cranberry phenolics onto different protein matrices with 50% of proteins (medium roast peanut or defatted soy flours) to protein matrices with over 70% of proteins (pea, soy or hemp protein isolates), a non-linear trend was achieved when protein amount and the adsorption capacity for phenolics were put in correlation [17]. In that study, cranberry phenolics had the highest affinity towards defatted soy and medium roasted peanut flours as well as towards hemp protein isolate. Additionally, a mentioned non-linear trend was obtained in another study, which deals with the adsorption of blueberry anthocyanins on matrices with lower amounts of proteins such as corn flour (5.3%), brown rice flour (8.6%), white whole-wheat flour (13%) and defatted soy flour (47%) [21]. Generally looking, the reactivity of phenolics towards proteins is correlated with two main factors, one is the number of hydroxyl groups and the other is hydroxyl groups position in phenolics structure [32]. The binding capacity of some phenolics (quercetin, kaempferol, myricetin, flavone apigenin, chlorogenic acid, caffeic acid, gallic acid) towards soy protein revealed that among those phenolics, the highest affinity had gallic acid, followed by chlorogenic acid and quercetin [32]. Comparison of the binding capacity of chlorogenic acid, ferulic acid, gallic acid, catechin, quercetin and apigenin towards albumin and globulin was conducted. Authors have determined that quercetin and catechin had equal binding capacity towards albumin; it was lower than for chlorogenic and gallic acid but higher than for apigenin and ferulic acid. The binding capacity of quercetin towards globulin was lower than for chlorogenic acid, catechin and gallic acid, but higher than for apigenin and ferulic acid [33]. Different studies emphasized that covalent and/or non-covalent interactions can occur between phenolics and proteins [34,35,36]. Non-covalent ones include interactions through hydrogen bonds, hydrophobic association, van der Waals forces and electrostatic attraction. However, as the most important non-covalent interactions for the complexation of proteins with phenolics, hydrophobic interaction and hydrogen bonds were pointed out [37]. Sui et al. [38] studied the binding of anthocyanins with soy protein isolate while increasing the amount of anthocyanins in the initial mixture and determined that the increase in binding of anthocyanins was proportional to their increase in the initial mixture. We obtained similar results in our study for the brown rice protein matrix that contained a higher protein content, which could be the reason for more pronounced hydrophobic interactions.

Numerous methods for evaluation of the antioxidant activity of foods, dietary supplements and nutraceuticals are available in the literature and can be applied. We selected DPPH, FRAP and CUPRAC methods, which are based on different mechanisms of action. The DPPH method is based on the reaction of radicals with hydrogen-donating antioxidants, which leads to the formation of the non-radical form. One of the characteristics of the DPPH radical is its selectivity in the reaction with hydrogen donors [39]. From our results, it can be observed that with the increase in quercetin amount, DPPH antioxidant activity increased; however, this increase was not proportional to the increase in quercetin amount on microparticles. Results of the other two methods that were used better followed the trend of quercetin amount on microparticles. In CUPRAC assay, the reduction of Cu(II) to Cu(I) by antioxidants is measured spectrophotometrically, while the reduction of Fe(III) complex to Fe(II) caused by the presence of antioxidants is assessed by FRAP [40,41]. According to our results, microparticles had a significantly higher capability of reduction of Cu(II) to Cu(I) than of Fe(III) to Fe(II).

Interactions between phenolics and proteins can cause a change in the denaturation temperature of the corresponding protein matrix. Usually, this parameter is used for the prediction of thermal stability of formulated protein/phenolic complexes [32]. When the denaturation temperature of formulated protein/phenolic microparticles is higher than the denaturation temperature of the corresponding protein matrix, the formulated microparticle is more stable than the protein matrix and vice versa [42]. From our results, it can be concluded that the adsorption of quercetin on the brown rice protein matrix resulted in its thermal stabilization. However, quercetin affected the almond protein matrix differently, i.e., it caused the decrease of thermal stability. Results of the other studies showed positive, negative or no effect on proteins stability depending on types of proteins and phenolics. The increase in thermal stability was achived when cinnamic acid was adsorbed on the almond protein matrix, while adsorption of the same phenolic acid onto the pea and pumpkin protein matrix had the opposite effect [27]. Green tea polyphenols caused a decrease of the thermal stability of β-lactoglobulin and egg albumen [42,43]. Complexes of soy protein with quercetin, myricetin or phenolic acids had higher stability than protein alone, while flavone, apigenin or kaempferol did not affect the stability of the mentioned protein [32]. The adsorption of chlorogenic acid on lysozyme, bovine serum albumin and α-lactalbumin also caused the increase of thermal stability of those proteins [44,45]. A conjugate of (−)-epigallocatechin gallate and zein had higher thermal stability than pure zein, while conjugates of zein and quercetagetin or chlorogenic acid had similar denaturation temperatures as protein alone [46]. A decrease of thermal stability was also observed upon the adsorption of raspberry juice phenolics onto brown rice proteins [26].

Structural changes of proteins upon adsorption of phenolics depend on protein structure that can be proved by a recoding of the IR spectra of microparticles and their comparison with the protein matrix. On both types of microparticles (AP/Q and RP/Q), a difference at the amide A region (3500 cm^−1^ to 3000 cm^−1^ assigned to N-H stretching coupled with hydrogen bonding) was observed, which could be an indication of non-covalent interactions between proteins and phenolics, i.e., indication of hydrogen bonding or hydrophobic association [46]. These interactions were probably involved in the adsorption of quercetin onto two selected matrices in our study. Alternation in IR spectra of proteins in the region of 3000 to 2800 cm^−1^ (assigned to CH_2_ antisymmetric and symmetric stretching vibrations) are an indicator of the existence of hydrophobic contact in the protein/phenolic complexes [47]. Hasni et al. [47] determined these changes in the complexes between α-caseins and β-caseins with tea phenolics. Based on the shifting of the protein antisymmetric and symmetric CH_2_ stretching vibrations, they proposed the existence of hydrophobic interactions throughout phenolics rings and hydrophobic pockets in caseins. Considering these results, we can also assume that those hydrophobic interactions occurred between the quercetin and protein matrices used in this study. In RP/Q microparticles, we also observed a shift of bands in this region that can be connected to hydrophobic interactions, as proposed before. On the other hand, on IR spectra of AP/Q microparticle, a change in intensity of bands in this region occurred, i.e., upon adsorption of quercetin, a decrease of the band intensity of protein occurred, suggesting a similar mechanism of interactions. Additionally, these different changes in protein matrix structure could be explained by the fact that the brown rice protein matrix had higher (85%) while almond had lower (50%) protein content; therefore, shifting was more pronounced on RP/Q microparticles. In addition, according to the quercetin amount adsorbed on protein matrices, these interactions were more pronounced for RP/Q microparticles since they contained a higher amount of this phenolic compound and its adsorption was proportional to initial concentration. Probably, these hydrophobic interactions as well as the higher amount of adsorbed quercetin were the reason for the improvement of thermal stability of RP/Q microparticles. Additionally, there was no change in IR spectra in Amid I structure, which could also lead to higher thermal stability of RP/Q complexes.

## 5. Conclusions

Functional ingredients with a broad range of applications are becoming more and more popular. Two plant-based protein matrices, brown rice and almond, were chosen for complexation with quercetin. The brown rice protein matrix had a higher affinity for quercetin than almond; thus, it would be a more efficient carrier of this phenolic compound. Additionally, it was observed for the brown rice protein matrices that with the increase in quercetin concentration in the initial mixture, the amount of quercetin on microparticles proportionally increased. This trend was not observed for the almond protein matrix. Additionally, brown rice protein microparticles were thermally more stable in comparison to the corresponding protein matrix, while the reverse trend was determined for almond protein microparticles.

## Figures and Tables

**Table 1:** Amount of quercetin (mg/100 g) and antioxidant activity (µmol TE/100 g) of protein/quercetin microparticles.

Microparticles	Q Amount	DPPH	CUPRAC	FRAP
Almond protein matrix				
AP/Q_1	60.18 ± 0.17 ^a^	29.38 ± 0.95 ^a^	106.39 ± 0.93 ^b^	0.66 ± 0.04 ^a^
AP/Q_2	98.38 ± 1.21 ^b^	30.63 ± 0.21 ^a^	125.42 ± 1.91 ^c^	1.02 ± 0.01 ^b^
AP/Q_5	196.34 ± 1.45 ^d^	32.18 ± 0.00 ^b^	192.75 ± 1.14 ^e^	2.34 ± 0.08 ^c^
Brown rice protein matrix				
RP/Q_1	108.24 ± 1.75 ^c^	31.12 ± 0.47 ^a,b^	84.38 ± 1.91 ^a^	1.20 ± 0.02 ^b^
RP/Q_2	226.50 ± 3.17 ^e^	34.22 ± 0.12 ^c^	161.59 ± 1.19 ^d^	2.78 ± 0.04 ^c^
RP/Q_5	506.98 ± 0.42 ^f^	40.40 ± 0.52 ^d^	414.52 ± 1.03 ^f^	8.35 ± 0.19 ^d^

Q—quercetin; AP—almond protein matrix; RP—brown rice protein matrix; 1, 2 and 5—represent concentration (mM) of initial quercetin solution; data in one column labeled with different letters statistically differ.

**Table 2:** Temperatures of denaturation (T_d_) of protein matrices and protein/quercetin microparticles.

Samples	T_d_ (°C)
Almond protein matrix	
100%	85.24 ± 0.07 ^b^
AP/Q_1	83.71 ± 0.28 ^a^
AP/Q_2	83.27 ± 0.25 ^a^
AP/Q_5	83.24 ± 0.29 ^a^
Brown rice protein matrix	
100%	85.26 ± 0.05 ^b^
RP/Q_1	85.78 ± 0.12 ^c^
RP/Q_2	86.70 ± 0.14 ^d^
RP/Q_5	86.72 ± 0.22 ^d^

Q—quercetin; AP—almond protein matrix; RP—brown rice protein matrix; 1, 2 and 5 represent concentration (mM) of initial quercetin solution; data in column labeled with different letters statistically differ.

## Data Availability

Data are contained within the article.
